# Mechanism of metastasis to the spermatic cord and testis from advanced gastric cancer: a case report

**DOI:** 10.1186/s12876-020-01269-0

**Published:** 2020-04-20

**Authors:** Soyoung Park, Sung Kyoung Moon, Joo Won Lim

**Affiliations:** Department of Radiology, Kyung Hee University Hospital, School of Medicine, Kyung Hee University, 23, Kyung Hee Dae-ro, Dongdaemun-gu, Seoul, 02447 Republic of Korea

**Keywords:** Gastric cancer, Metastasis, Spermatic cord, Testis, Paraaortic lymph node, Aortocaval lymph node

## Abstract

**Background:**

The spermatic cord and testis are very rare sites for metastasis from gastric cancer. Although several mechanisms have been suggested to explain this unusual metastasis, the actual mechanism remains unclear. We report a case of right spermatic cord and testicular metastasis, review its imaging findings, and suggest a mechanism of tumor spread.

**Case presentation:**

A 61-year-old man complained of a palpable mass in the right inguinal area. He had been treated with distal gastrectomy with chemotherapy for advanced gastric cancer 5 years ago. Computed tomography, ultrasound, and magnetic resonance imaging showed a mass surrounding the right spermatic cord, involving the right testis. Another mass was observed in the aortocaval space, presumed to be a metastatic lymph node. The imaging features of the right testicular lesion were different than those of the primary testicular cancer. The lesions at both sites showed similar radiologic features of abundant internal necrosis, which is consistent with metastatic lesions. Pathology confirmed metastatic adenocarcinoma. He underwent a series of chemotherapy sessions, and all metastatic masses had partially decreased in size at the 5-month outpatient follow-up.

**Conclusions:**

The imaging features of testicular mass and spermatic cord involvement are important clues for accurate differential diagnosis of metastasis from other primary tumors in patients with a history of stomach cancer. This unusual metastasis can be explained via retrograde tumor spread along the lymphatic channels in terms of concurrent aortocaval lymph node metastasis. A suspicion of metastasis should not be overlooked, even if a patient has undergone curative treatment, including surgery and adjuvant chemotherapy, many years ago.

## Background

Gastric cancer is one of the most common malignancies worldwide, especially in east Asia [1]. The most common metastatic organs for gastric cancer include the liver, peritoneum, lung, and bone marrow [2]. The spermatic cord and testis are very unusual sites of metastasis. Only 30 cases of metastatic tumors from gastric cancer involving the male genital tract, including the spermatic cord, vas deferens, testis, epididymis, and scrotum, have been reported since 1955 [3]. Their mechanisms have not been clearly explained yet. Here, we report a case of metastatic tumor in the right spermatic cord and testis from gastric cancer, review its imaging findings, and suggest a mechanism of tumor spread.

## Case presentation

A 61-year-old man underwent subtotal gastrectomy with Billroth I anastomosis and received chemotherapy (XELOX) for advanced gastric cancer. Five years later, he visited a hospital with a palpable mass in the right inguinal area and underwent computed tomography (CT).

**Fig. 1:**
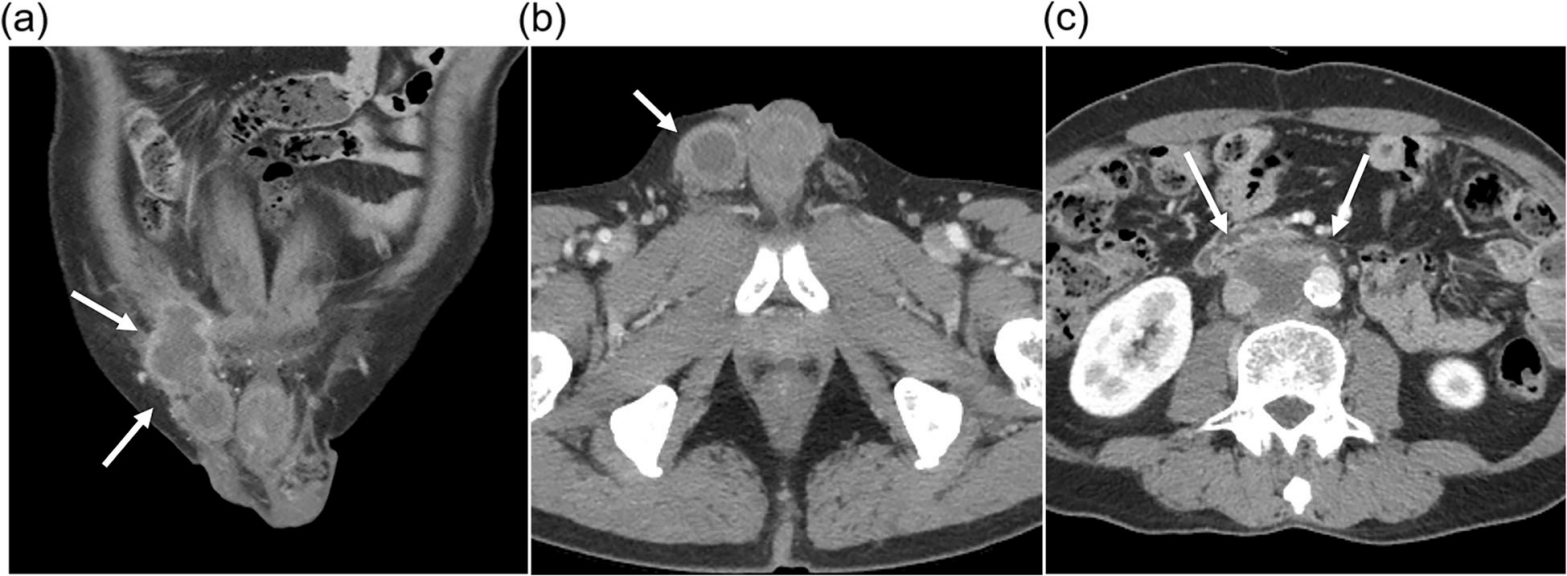
Computed tomography images. **a** An irregular elongated mass (arrows) along the right inguinal canal and right external iliac vessels (**b**) A round mass (arrow) at the lower portion of right inguinal canal, probably arising from the right testis. **c** Another mass (arrows) at the aortocaval space at the L3 level indicating metastatic lymphadenopathy

**Fig. 2:**
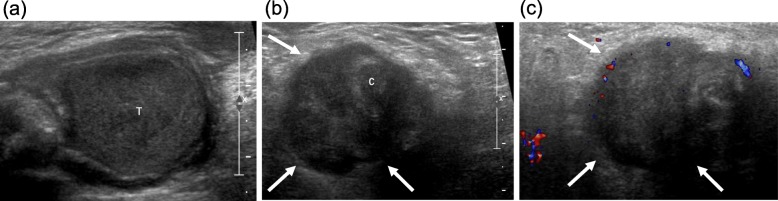
Scrotal ultrasonography images. **a** Right testis (T) with normal contour but decreased echogenicity. **b** A hypoechoic mass (arrows) above the right testis, surrounding the spermatic cord (C). **c** No increased blood flow in the right testis and the hypoechoic mass (arrows) on color Doppler ultrasonography (CDUS)

**Fig. 3:**
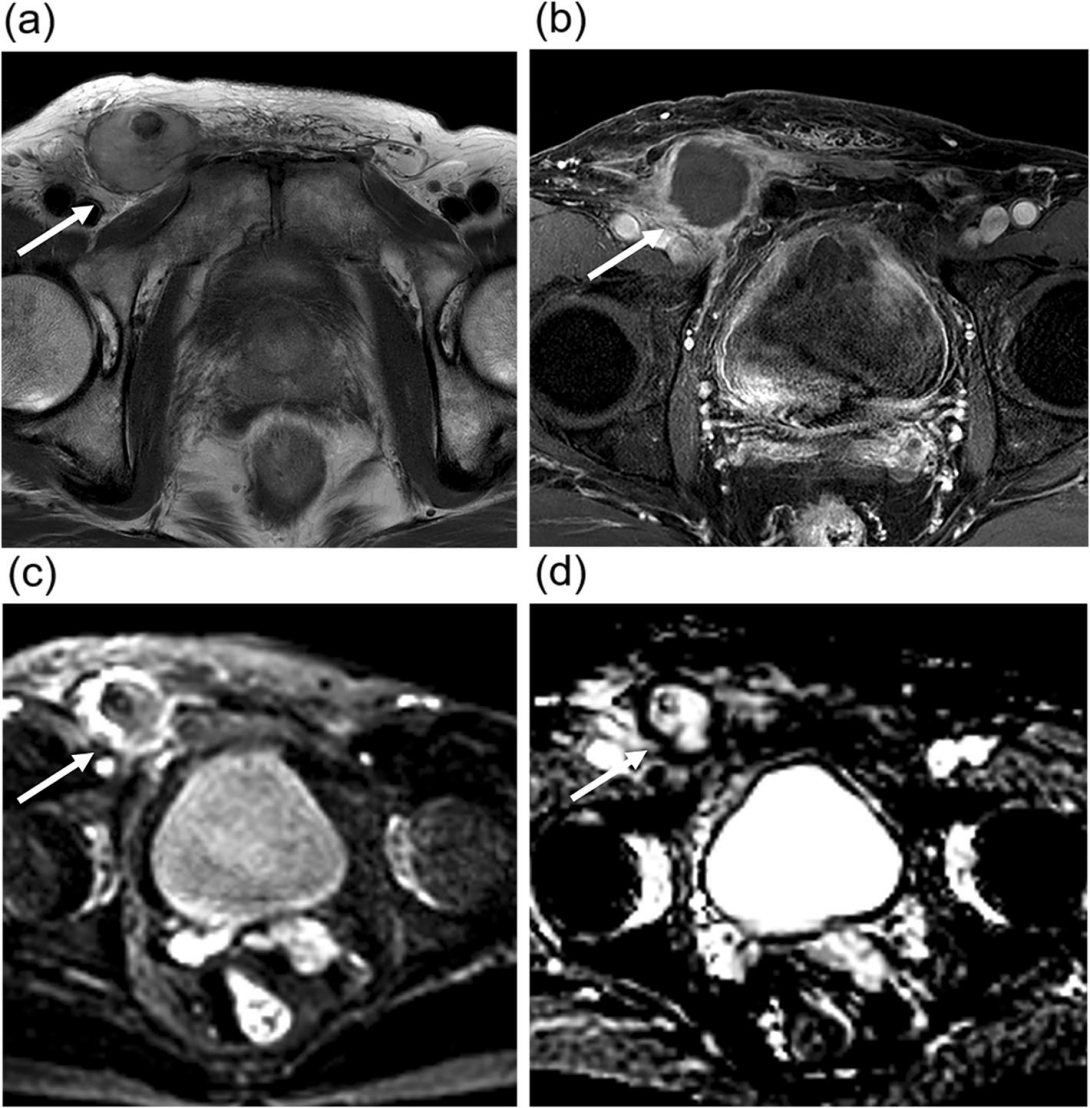
Magnetic resonance images. **a** A hyperintense mass (arrow) surrounding the hypointense spermatic cord in T2-weighted images. **b** Peripheral rim enhancement of the mass (arrow) surrounding the spermatic cord in a contrast-enhanced fat suppression T1-weighted image, (**c-d**) Diffusion restriction along the mass periphery (arrows) of the mass surrounding the spermatic cord on diffusion weighted image (b = 1000) and apparent diffusion coefficient (ADC) map

CT showed a bilobed mass with 2 components of a round portion, measuring 2.5 cm, at the caudal aspect of the right inguinal canal, and an additional 6.6-cm irregular elongated portion straddling the right inguinal canal and right pelvic cavity along the right external iliac vessels (Fig. 1a). The right scrotal sac was empty and without the normal right testis. The 2.5-cm round mass appeared to be the right undescended testis (Fig. 1b). It was presumed to be affected by the right inguinal canal mass rather than the primary testicular cancer, with extension to the nearby structures because of its relatively preserved size and contour. There was another 4-cm mass in the aortocaval space at the third lumbar spine level, which was presumed to be metastatic lymphadenopathy (Fig. 1c). Masses in the right inguinal area and aortocaval space had similar imaging characteristics. Both lesions showed peripheral rim enhancement with internal low attenuation of a necrotic portion. Scrotal ultrasonography (US) revealed the right testis at the caudal aspect of right inguinal canal, similar to the CT findings. The right testis appeared to maintain a relatively normal contour on US as well as on CT. However, it had less echogenicity than the normal left testis (Fig. 2a). The right inguinal and pelvic masses above the right testis appeared as a hypoechoic mass partially surrounding the spermatic cord, which was correlated with the right inguinal canal mass on CT (Fig. 2b). There was no increased vascularity on color Doppler ultrasonography (CDUS) (Fig. 2c). On magnetic resonance (MR) T2-weighted images (T2WI), the mass surrounding the spermatic cord and involving the right testis showed high signal intensity (Fig. 3a). Peripheral rim enhancement and diffusion restriction were shown on contrast-enhanced T1-weighted images (CE-T1WI) and diffusion-weighted imaging (DWI) with high b-value, respectively (Fig. 3b-d). Most central contents had no enhancement and no diffusion restriction, indicating mostly necrotic portions. Serum alpha fetoprotein (AFP) and beta-human chorionic gonadotrophin (β-hCG) levels were normal.

Differential diagnoses for the right inguinal and aortocaval masses included metastatic lesion, testis cancer, lymphoma, or sarcoma originating in the inguinal canal. However, primary testicular cancer was less likely, as the right testis maintained its relatively normal shape and contour. The epicenter of the mass seemed to be the inguinal canal mass rather than the right testis, which is different from the usual primary testicular cancer. In addition, it was presumed that the right testis moved upward as the mass in the right inguinal canal affected the spermatic cord, because the patient said the right testis had recently disappeared from the right scrotum. Lymphoma usually presents as a homogeneous soft tissue mass without necrosis, unlike as a mass containing mostly necrosis, as seen in the present case. Primary sarcomas rarely develop in the inguinal canal. It usually originates from the outside of the testis such as spermatic cord, scrotal wall, or epididymis. Sarcomas are a heterogeneous group of tumors arising from the embryonic mesoderm and the most commonly reported as liposarcoma, leiomyosarcoma, carcinosarcoma, and rhabdomyosarcoma in the genitourinary tract [4]. Among those, well-differentiated liposarcoma can be excluded from the differential diagnosis of our case because it usually appears a large mass of fat attenuation with septa on CT scan and high-signal intensity on both T1- and T2-weighted images with little enhancement on MRI. Imaging results for other types of sarcomas are nonspecific, and biopsy is usually required for a definitive diagnosis [5]. Nevertheless, we still thought that the inguinal canal mass was less likely to be sarcoma because of the presence of another aortocaval mass, which suggests a metastatic lymph node. The prevalence of nodal metastasis is low in soft-tissue sarcomas [6]. Most subtypes of sarcomas spread and recur by direct invasion rather than lymph node metastasis; thus, elective lymphadenectomy is rarely required [7]. As the patient had a history of stomach cancer, metastasis to the retroperitoneal lymph nodes was possible, even though the external iliac and inguinal area and the right testis are uncommon metastatic locations. The similar imaging features of these masses with abundant internal necrosis was consistent with metastasis.

Right orchiectomy was planned initially. However, as imaging findings strongly suggested metastasis, US-guided biopsy was performed. Pathology of the US-guided biopsy specimen from the right inguinal mass revealed metastatic adenocarcinoma with necrosis. The primary sites were considered to be the pancreatobiliary tract and stomach. The patient received multiple cycles of chemotherapy (FOLFOX), and all metastatic masses decreased in size at the 5-month outpatient follow-up.

## Discussion and conclusions

Spermatic cord and testis are rare sites of metastasis. In our case, stomach cancer was the primary lesion of the metastatic mass at the spermatic cord, based on the pathologic report and the patient’s history of advanced gastric cancer. The stomach is known to be the most common primary site of spermatic cord metastasis, especially in east Asia [8]. The colon, liver, kidney, pancreas, lung, and prostate have been reported as other primary origins of spermatic cord metastasis [9]. Testicular metastasis is very rare, with an incidence ranging from 0.02 to 2.5% [10].

The mechanism of metastasis from stomach cancer to the spermatic cord or testis remains unclear. Several pathways, such as lymphatic, vascular, hematogenous, and peritoneal, have been suggested [10, 11]. The main proposed routes are the lymphatic and/or vascular pathways [3]. Based on our findings, we also suggest the lymphatic route for metastasis to the spermatic cord from the gastric cancer. Lymphatic spread is frequently found in gastric cancer patients, even in early gastric cancer, which is attributed to the abundant perigastric lymphatic channels [12]. In our patient, there was an additional mass in the aortocaval space at the third lumbar spine level, considered to be conglomerated metastatic lymph nodes. The paraaortic lymph node is one of the final gastric lymphatic drainage stations (number 16) according to the gastric nodal stations defined by the Japanese gastric cancer association [13]. Lymph from the upper third of the stomach flows through lymphatic channels along the left gastric, posterior gastric, splenic, and left inferior phrenic arteries. Lymph from the lower third of the stomach flows through lymphatic channels along the common hepatic artery and the root of the superior mesenteric artery, which drains into the hepatoduodenal and retropancreatic lymph nodes. These lymphatics ultimately run into the paraaortic lymph nodes [14]. Furthermore, a paraaortic lymph node pathway connects the spermatic cord and the aortocaval lymph nodes. It is known that the main route of metastases from testicular carcinoma bypasses the pelvic lymph nodes. The lymphatic vessels of the testes lying with the gonadal blood vessels ascend through the spermatic cord, pass through the anterior aspect of psoas muscle, and end in the paraaortic and paracaval nodes at the level of the renal hilum. A retrograde metastatic spread occurs downward through these lymph nodes [15]. Therefore, the spermatic cord can also be a site of metastasis from stomach cancer through these lymphatic pathways in a retrograde manner. In terms of overlaps of the metastatic routes between stomach and testicular cancers, the imaging features can be the most important clue for the differential diagnosis of primary sites. Most forms of testicular cancer are germ cell tumors and occur in young populations (the second to fourth decades of life), irrespective of whether they are seminomas or non-seminomatous germ cell tumors (NSGCTs). It manifests as massive enlargement of the testis and a mostly solid mass with various proportions of necrosis on imaging studies [16], which is quite different from that seen in the present case.

Metastatic lesions developed more than 5 years after surgery for stomach cancer in our patient. Recurrence of gastric cancer often occurs early, commonly within the first 2 years after gastrectomy; recurrence after 10 years is extremely rare [17]. There is also a difference in the recurrence pattern depending on the time of relapse. In the study by Moon et al. [18], which was a 15-year follow-up study of advanced gastric cancer after gastrectomy and adjuvant chemotherapy in 500 patients, distant metastasis was the predominant recurrence pattern 5 years post-gastrectomy, whereas peritoneal carcinomatosis was the predominant recurrence pattern in patients showing relapse within 5 years of gastrectomy. Our patient received adjuvant chemotherapy (XELOX) after distal gastrectomy, which implies advanced stage of disease with lymph node metastasis. Although the spermatic cord and testis are rare metastatic sites, metastasis should be always considered based on the patient’s clinical information and underlying disease. A suspicion of metastasis should not be overlooked, even when many years have passed since treatment for stomach cancer, especially for advanced stage disease.

Metastatic tumor of advanced stomach cancer in the male sex cord has been known to have poor prognosis. A recent study reported an 1-year overall survival rate of 38.7% [3]. In the report by Kim et al. [19], 2 patients were stable at the 26- and 20-month follow-ups without recurrence after orchiectomy or radiation therapy; both these patients presented with late-onset metastasis (7 and 6 years). In most previous cases, the patients underwent radical orchiectomy for metastatic tumors in the spermatic cord [3, 9–11]. However, systemic chemotherapy is necessary for preventing distant metastasis from gastric cancer rather than orchiectomy alone for curative treatment. In our patient, there was no indication of curative resection owing to aortocaval lymph node metastasis. He has been receiving chemotherapy since detection of the metastatic lesion with preservation of the testis. The patient has been undergoing outpatient follow-up in stable condition, and the sizes of the metastatic tumors at the spermatic cord and aortocaval space had decreased partially at the 5-month follow-up.

In conclusion, gastric cancer can metastasize to the spermatic cord and testis via lymphatic channels in a retrograde manner. This metastatic pathway overlaps with that of metastasis from primary testicular cancer. However, adequate imaging studies can discriminate the primary sites (stomach cancer vs. testicular cancer). If imaging findings strongly suggest metastasis, simple US-guided biopsy can be used for confirmation rather than orchiectomy before systemic chemotherapy. Lastly, metastasis should always be considered in patients with a history of stomach cancer, even if the patient has undergone curative treatment, including surgery and adjuvant chemotherapy, many years ago.

## Data Availability

The detailed information and images of the present case are available from the corresponding author on reasonable request.

## Abbreviations

CT: Computed tomography
US: Ultrasonography
CDUS: Color Doppler ultrasonography
MR: Magnetic resonance
T2WI: T2-weighted images
CE-T1WI: Contrast-enhanced T1-weighted images
DWI: Diffusion-weighted image
AFP: Serum alpha fetoprotein
β-hCG: Beta-human chorionic gonadotrophin
NSGCT: Non-seminomatous germ cell tumor
ADC: Apparent diffusion coefficient
